# The silence of opioids-dependent chronic pain patients: A text mining analysis from sex and gender perspective

**DOI:** 10.1371/journal.pone.0319574

**Published:** 2025-03-18

**Authors:** Claudia Carratalá, Laura Agulló, Patricia Carracedo, Noelia Serrano-Gadea, Mónica Escorial, Elena López-Mañogil, Pau Miró, Sónia F. Bernardes, Ana M. Peiró

**Affiliations:** 1 Clinical Pharmacology, Toxicology and Chemical Safety Unit, Institute of Bioengineering, Miguel Hernández University, Elche, Spain; 2 Neuropharmacology Applied to Pain (NED), Clinical Pharmacology Unit, Dr. Balmis General University Hospital, Alicante Institute for Health and Biomedical Research (ISABIAL), Alicante, Spain; 3 Department of Statistics and Operational Research, Universitat Politècnica de València, Alcoy Campus, Alicante, Spain; 4 Faculty of Psychology, Universidad Católica de Murcia, Murcia, Spain; 5 Iscte-University Institute of Lisbon, Centre for Social Research and Intervention (CIS-Iscte), Lisbon, Portugal; University of New Mexico Health Sciences Center, UNITED STATES OF AMERICA

## Abstract

Existing evidence indicates sex-related differences in Prescription Opioid Use Disorder (OUD) in Chronic Non-Cancer Pain (CNCP). However to date, there is scant evidence for other socioeconomic factors in these differences. Our aim was to enquire about the influence of gender and drug copayment of OUD narratives by the text mining analysis. A prospective mixed-methods study was designed and performed at Pain Unit (PU) including 238 real world patients with CNCP divided in controls (n = 206) and OUD cases (n = 32) due to DSM-5 diagnosis Variables related to pain, sleep, mental and health status were collected in together with sex and gender interaction, in pain status, along 30-45 min face-to-face interviews. Sex differences were observed due to women’s significantly older ages, with a stronger impact on mental health, and an even stronger one for the OUD women. Globally, OUD cases were more unemployed vs the CNCP controls, and on a significantly higher median opioid daily dose of 90 [100] mg/day. Although OUD participants did more social activities, they tended to use less vocabulary to express themselves regardless of their sex, gender role or economic status. In contrast, the CNCP participants presented more differences driven by their incomes, with “limited” being the most discriminating word for those on low income, followed by “less” and “help”. Here, the most significant word of CNCP women was “husband”, followed by “tasks”. In contrast, gender reproductive roles shared similarities in both sexes, being one of the most discriminatory words “help”. The data show that OUD patients seem to have a marked influence of OUD on poorer lexicon and simpler narrative, together with a significant impact of socioeconomic factors on the CNCP narratives. The conclusion suggests to extend the research to better understand the effect of sex, gender and socioeconomic status in CNCP especially on OUD women’s health.

## Introduction

The drug addiction stigma is a significant barrier in Chronic Non-Cancer Pain (CNCP) management [1,2]. There are well-established risk factors for opioid use disorder (OUD), such as younger age, history of substance dependence and/or mental illness, and higher opioid doses [3,4]. Although sex and gender differences in CNCP have been previously investigated in this context [5], the literature contains very few studies with clinical translation in OUD management. Understanding attitudes towards the individuals who use opioids is a crucial matter to tackle negative perceptions of opioid use during medical visits improving adherence and tolerability [6].

Data have revealed different substance use patterns, health and social functioning between men and women [7]. Recent studies suggest sex-related differences because women are more likely to undergo inadequate pain management [8], together with more difficulty with accessing pain care [9]. These sex-related differences highlight areas for improved intervention, especially when OUD is detected [10]. This potential sex and gender influence could be used to develop an integrative treatment regimen according to individual needs in relation, i.e., to gender roles.

In principle, the term “sex” refers to biological differences between men and women, specifically reproductive organs and their functions, while the term “gender” refers to the social context in which people live and which contributes to a subjective sexual identity, masculine or feminine. It is based on cultural norms and specific to a historical era and, therefore, is constantly changing, influencing everyday actions, societal expectations and experiences [11]. These are the behaviors that men and women exhibit in private and public realms. Globally, they are socio-cultural expectations that apply to individuals according to their assignment to a sex category [12]. In this study, productive (e.g., paid- workers) or reproductive roles (e.g., childcare and parenting assistance or relationship) gender roles were considered to enhance understanding of the identification for this challenging and growing population [13]. Moreover, sex-related differences and gender roles are intersected by other social determinants, including income, education, or occupation. All are critical factors that contribute to ongoing health disparities between sexes [14] in pain management [15].

As part of health systems research, a gender analysis seeks to understand how gender power relations create inequities in access to resources, the distribution of labor and roles, social norms and values, and decision making [16]. In fact, women have traditionally experienced heightened vulnerability to the adverse medical and social consequences of opioid dependence as a result of biological sex-related characteristics and socially defined gender roles’ [17].

Nowadays, the innovative text mining analysis, which combines data mining, linguistics and computer science [18], has proven to be a useful tool to extract information from unstructured data, involving the detection of knowledge from textual data. It is used worldwide in many settings. In healthcare, it has been used to identify adverse drug events, help physicians make diagnoses, and informed treatment decisions [19–22]. This could allow us to obtain qualitative information from unstructured texts as individuals’ interviews. This could help us to understand how sex and gender influence patients’ narratives about their CNCP experiences [23], which are often crucial to gain access to opioid prescriptions [24], and thus a potential higher risk to OUD.

For this reason, our main goal was to use text mining to: study the influence of sex, gender and drug copayment in OUD narratives by the text mining analysis. 1) Evaluate if sex and gender differences could be found between CNCP patients’ narratives. 2) Evaluate if sex and gender differences could be found between OUD patients’ narratives and control subjects. 3) Figure out whether these differences could be associated with other socioeconomic variables, such as drug copayment.

## Materials and methods

### Study design and participants

A prospective mixed-methods study was designed and performed at the Pain Unit (PU) of the Alicante Health Department–Dr. Balmis General University Hospital in Spain. The recruitment period for this study was from October 1st 2021 to September 30th 2023 and it included 238 patients with CNCP (71% women; 13% cases with OUD) to provide quantitative and qualitative information. The inclusion criteria were adults (≥18 years) with CNCP who required opioid analgesic treatment and signed informed consent. The subset of patients with overlap of CNCP and OUD were evaluated with a specific questionnaire to assess pain. Moreover, we also have available different tools to explore pain. In the case of OUD, these patients are first evaluated in our unit with the DSM-5 [25]. Positive cases for OUD were classified and referred to a consult with a multidisciplinary team composed of a specialized doctor, a pharmacologist, a psychologist and a primary care doctor.

The patients with oncologic pain or any psychiatric disorder (depression and anxiety) that could interfere with properly performing this study were excluded. Other chronic pain syndromes of unclear pathophysiology, such as fibromyalgia or neuropathic pain, such as painful polyneuropathy, postherpetic neuralgia, trigeminal neuralgia, and post-stroke pain, were not included because opioids are not recommended for some of these conditions [26].

### Ethics statement

This study was approved by the Ethics Committee Board of the Dr. Balmis General University Hospital of Alicante (code SESGEN text mining: PI2023-021). All subjects gave verbal and signed informed consent before participating in interviews. Confidentiality of all the information was guaranteed. The study was done in accordance with the Declaration of Helsinki regarding research involving human subjects.

### Procedure and data collection

A consecutive sampling method was used, including all patients who attended consultations during the data collection period according to their scheduled medical appointments. The researchers and interviewers reviewed the schedule of the PU-appointed patients one day a week (usually Thursdays) and prepared the questionnaires and informed consents. When patients met the inclusion criteria, they were informed by the PU healthcare team about the purpose of the study. Then, any interested individuals were asked by the research staff to sign an informed consent. All variables were collected, including an OUD diagnosis following the DSM-5 criteria [25]. If necessary, the participants’ clinical data were completed using electronic health records (EHRs), which allows medical diagnoses, outcomes and medication use to be reviewed.

#### Clinical variables.

Several demographic characteristics, such as age, sex (women, men, non-binary people), employment (working, retired, work disability, unemployed or homemaker) were firstly registered (S1 Table). A Global Pain State questionnaire was used to evaluate pain intensity and relief during the interviews. Pain intensity and pain relief were measured using the Visual Analogue Scale (VAS) [27]. Both consist of a horizontal line ranging from 0 (lowest) to 100 mm (highest), where patients point out their pain intensity or pain relief on the line.

The EuroQol-5D-3L scale was used to evaluate quality of life. It consists of a VAS (vertical line from 0 the worst imaginable health status to 100 mm the best imaginable one) where patients indicate their current health status. Health utility status was calculated (0 death to 1 perfect health) based on five health dimensions (mobility, self-care, usual activities, pain/discomfort, anxiety/depression) with five severity levels on each dimension (none, mild, moderate, severe, and unbearable to perform or extreme problems; 53112 reg. number available at https://euroqol.org/) [27].

The Short Format Health Survey 12 (SF12) was used to measure health status. It consists of a 12-item questionnaire with a mental health (MCS, 0-100 scores) and a physical health score (PCS, 24-57 scores 0-100 scores) with a mean of 50 and a standard deviation of 10 in the general US population [28].

The Hospital Anxiety and Depression Scale (HADS, 0-21 scores) was used to measure psychological health. Scores were classified as negative (normal range, <  7), doubt (suggestive of mood disorder, 8 - 10) and case (probable presence of mood disorder, >  11) [29].

The 30-item validated “Impairment and Functioning Inventory” (IDF-R) related scale was used to measure pain interference in four life areas: Household Activities (11 items), Independent Function (7 items), Leisure Activities (4 items), and Social Activities (5 items). Additionally, there were two final scores, such as: Functionality level (0-108, from 0 times to activity done, i.e., “Have you driven a car?” to more than 10 times last week) and Impairment level (0-27, from 0 (no impairment) to 1 (yes)) (S2 Table) [30].

The Sleep Scale from the Medical Outcomes Study (MOS-SS) was used to measure sleep quality. It consists of a 9-item questionnaire that is self-administered and takes 2–3 minutes to complete. The sum of the scores of the items and domains becomes a numerical scale of 0–100 in all the items. Two exceptions are contemplated: the score of the item “quantity” ranges between 0–24 and the score of the item “adequacy of sleep” ranges between 0–1. Higher scores indicate worse sleep problems [31].

#### Pharmacology and use of hospital resources.

The use (yes/no) of simple analgesics (i.e., paracetamol and metamizole), non-steroidal anti-inflammatory drugs (NSAIDs), opioids (i.e., tramadol, codeine, fentanyl, oxycodone, tapentadol, buprenorphine, morphine, hydromorphone, methadone), along with immediate release opioids, were recorded. In different opioid combinations, the oral morphine equivalent daily dose (MEDD, mg/day) was estimated with available references [32].

In Spain, medical care (primary, specialized and hospital care) are completely free and universal. However, pharmaceutical copayment is the patient’s contribution to pharmaceutical services after Spanish Royal Decree Law 16/2012 came into force. This decree states that workers have to pay 40%, those with HIV and chronically ill patients pay 10%, and pensioners, disabled individuals and those with work-related illnesses are exempt. A 40% of copayment is fixed for annual incomes up to 18,000 euro. This percentage was fixed to classify low (>40%) or high copayment [33].

#### Quantitative and qualitative gender information.

Three trained interviewers conducted face-to-face interviews that lasted 30-45 minutes being blinded to the patient’s OUD status. The interviewers divided the weeks supporting the clinical consultation, in a variable way. The evaluator conducted the process as an interview, asking each question aloud and recording each participant’s responses verbatim. This approach allowed for the capture of the subjects’ exact verbal expressions, facilitating a detailed textual analysis of individual responses.

All the patients were self-reported as cis (“female” or “male”: the sample included no non-binary person) and a consecutive number was assigned. Quantitative information about gender roles assessed or gender identity was obtained by 15 questions. These questions were based on sex and gender roles studies [34]. Here questions 2-6 were related to gender identity (female/male), while gender roles were related to questions 7 (work), 9 (domestic responsibilities), 11-12 (partner relationships) and 13 (family) (S1 Questionnaire) [34]. Self-reported gender roles were reproductive role (childbearing and caring for children), unpaid domestic tasks to maintain homes (cooking, fetching water, cleaning, washing clothes and similar) and productive role, which is work done to produce goods and services for consumption or trade (S3 Table) [35]. These gender roles are, respectively, associated in society with men and women in a stereotypical manner [36]. The percentage of affirmative answers to each question was compared between sexes. Finally, a group of testimonials selected from the different questions, and clearly associated with gender conflict, were separately analyzed. Some of the speeches of participants’ testimonials were selected to illustrate differences. Authors did the first interviews’ statements’ translation from Spanish to English by pairs. The final check testimonials (Spanish and English translation) from the database were then checked by a native English speaker.

### Text mining analysis

Text mining is a variation in the field called data mining, which attempts to find interesting patterns from large databases. The objective is to discover unknown high-quality information from a text. The first step of this technique is to specifically obtain a textual document to process it, and to check its formatting and characters [37]. In this study the followed steps were to: 1. remove redundancies by ignoring cases; 2. delete punctuation marks; 3. remove digits at the end and delete commonly used stop words in a language that do not provide any information in the text analysis. Some examples in English are: that, then, the, a, an, and, among others. Next the processed document goes to the textual analysis phase to discover any important knowledge to be processed. Figures represent the most frequently used words (triangle) by the OUD diagnosed patients due DSM-5 criteria (yes/no) to describe their CNCP experience, due to sex, gender role or drug copayment.

### Statistical data analysis

A descriptive analysis of the continuous quantitative variables is presented as the mean and standard deviation (M ±  SD). The discrete variables are shown using their median and interquartile range (Med [IQR]). Categorical data is expressed as percentages (%). A correspondence analysis (CA) is used to describe any potential relations between variables [38]. This involves the normalization of the cross-table of frequencies so that cross-table entries can be represented in terms of the distance between dimensions in a low-dimensional space. The CA represents the rows and columns of a data matrix as points in a spatial representation called a map or a biplot [39,40]. The positions of points suggest facilitated interpretations of data content [41]. Each word is represented by a triangle, and its size indicates the frequency of the word and the distance to the closest one denotes the similarity between them [42]. The distance between words and the origin coordinates (0, 0) measures the quality and contribution to the representation. Therefore, the words that lie further from the origin are more discriminatory and well-represented in the biplot [43]. Proximity between terms (groups) indicates similarity (clusters).

## Results

The study included 238 patients (71% women), whose mean age was 62 (14) years old, 35% retired with low medication copayment (10%) and 91% with monthly incomes below 1,000 euro. As can be seen in Table 1, the mean and median are the same or similar in the three groups which indicates that the age variable is symmetrical. For this reason, the results of text mining have not been analyzed by age as it was not the objective of the study. However, this aspect has been highlighted as a reference variable like other sociodemographic factors.

**Table 1:** Socio-demographic and clinical characteristics of the CNCP patients by an OUD diagnosis (yes or not).

Outcomes (mean (SD))	Total (n = 238)	OUD (n = 32)	no-OUD (n = 206)
**Sex** (%, Women)	71	69	71
**Age** (years old)	62 [52,73]	63 [53,72]	62 [51,73]
**Drug Copayment** (%)	10 (10)	10 (10)	10 (10)
**Employment Status** (%)			
Working	16	6	18
Retired	35	34	35
Work disability	22	19	22
Unemployed	6	19	4
Homemaker	14	6	15
**Monthly Income** (%, €/month)			
< 1,000	91	78	93
≥ 1,000	9	22	7
**Pain Intensity** (0-100 mm)	80 [50,90]	85 [60,100]	80 [50,90]
**Relief Intensity** (0-100 mm)	40 [0,60]	30 [0,60]	40 [0,60]
**Quality of Life**			
**Health Utility Status** (0-1)	0.32 (0.35)	0.25 (0.28)	0.33 (0.35)
**VAS Quality of Life** (0-100 mm)	50 [30,65]	50 [10,50]	50 [30,70]
**SF12** (0-100 scores)			
Physical health	26 (9)	24 (6)	26 (9)
Mental health	40 (19)	35 (24)	41 (18)
**Anxiety** (HADS, 0-21)	7 (6)	10 (6)	7 (6)
Negative (%)	35	22	37
Doubt (%)	14	6	15
Case (%)	18	22	19
**Depression** (HADS, 0-21)	7 (6)	8 (12)	7 (6)
Negative (%)	42	25	44
Doubt (%)	13	6	14
Case (%)	15	19	14
**Sleep** (MOS-SS, 0-100 scores)			
Sleep Problems Index I (SLP6)	40 (37)	40 (38)	40 (37)
Sleep Problems Index II (SLP9)	43 (32)	39 (42)	43 (31)

VAS: Visual Analogue Scale; SF12: Short Format Health Survey 12; PCS: Physical Component Score; MCS: Mental Component Score; HADS: Hospital Anxiety and Depression Scale; MOS-SS: Medical Outcomes Study Sleep Scale.

Clinical status was similar in all the participants with mean severe pain intensity (71 (27) mm) and mild relief (37 (31) mm), which resulted in moderate quality of life (47 (25) mm) and a healthy utility status of 0.32 (0.35) scores. Most patients were classified as negative cases for both anxiety and depression; with similar rates of sleep problems. Table 1 shows the socio-demographic and clinical characteristics of the CNCP patients by OUD diagnosis.

The pharmacological data shown in Table 2 evidenced that the OUD participants were prescribed with a significantly higher MEDD of 90 (100) mg/day in front of the CNCP no-OUD group. This first group also used more % of opioids, except for Tramadol, which was consumed mainly by no-OUD. Around half the patients used other coadjuvants (neuromodulators, anxiolytics followed by antidepressants) being similar between both groups.

**Table 2:** Pharmacological characteristics of the Chronic Non-Cancer Pain (CNCP) patients by an Opioid Use Disorder (OUD) diagnosis.

Outcomes (mean (SD))	Total (n = 238)	OUD (n = 32)	no-OUD (n = 206)
**MEDD** (mg/day)	45 (60)	90 (100)	40 (60)
**Main Opioid** (%)			
Tramadol	29	13	32
Tapentadol	15	13	16
Fentanyl	13	25	11
Buprenorphine	13	19	13
Oxycodone	8	19	7
Morphine	3	9	1
**Analgesic coadjuvants** (%)			
Antidepressants	45	38	46
Neuromodulators	54	56	54
Anxiolytics	50	44	51

MEDD: Morphine Equivalent Daily Dose.

### Differences in narratives due to OUD

**Fig 1:**
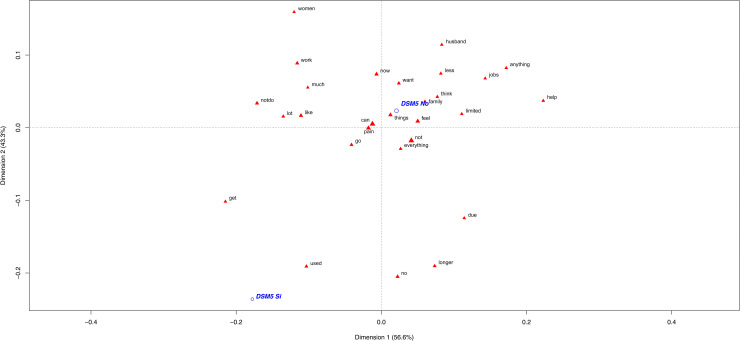
Most frequently used words by the OUD diagnosed patients (yes/no) to describe their CNCP experience. The first plane of the correspondence analysis with the most contributive terms of the Chronic Non-Cancer Pain (CNCP) patients according to their DSM-5 diagnosis (in blue, yes =  DSM-5 Si/ no =  DSM-5 No).

The OUD participants employed a scant, simple lexicon compared to the no-OUD CNCP patients (Fig 1). The words represented close to this first group did not clearly inform about how these patients described their pain. So it can be deduced that they *no longer do what they used to due to pain*. However, these words were neither discriminatory nor specific for the case group. One patient’s speech to illustrate this was: *“Everything has changed for me because I’m no longer who I used to be. I did certain things before, but not now”, “I want to do some things, but can’t”, “I try, but can’t due to pain”.*

Here it should be indicated that the no-OUD participants (86.5% of the sample included) used more words and a richer narrative. Thus, the controls used a more complex vocabulary with different terms to describe how pain impacts their performance in different areas of their life. Here the most significant words were *“husband”, “anything”, “help” and “tasks”*. Some testimonials to illustrate this were: *“I try to do tasks. When I can’t, my husband does them”, “If I don’t feel well. I don’t feel like doing anything”, “I ask my husband for help, but I want to do them myself”, “I feel I can’t do what I did before. I need help”* and *“It’s impossible for me to do certain tasks like picking something up off the floor”.*

### Sex and gender role differences in the CNCP narratives

#### Sex differences in patients’ narratives.

The tendency for the OUD patients (13.5% of total population) to use less vocabulary to express themselves was still remarkable regardless of their sex or gender role. Consequently, few differences in sex and gender roles among the OUD participants could be identified. As an example, one woman said *“I used to see myself in another type of life, a more optimistic life”* and *“I used to walk and manage myself, but no more”.* Men mentioned that *“I used to be very participative and now I can’t”* and *“I used to be more lively. Now I’m more agitated, depressed and sensitive”*.

**Fig 2:**
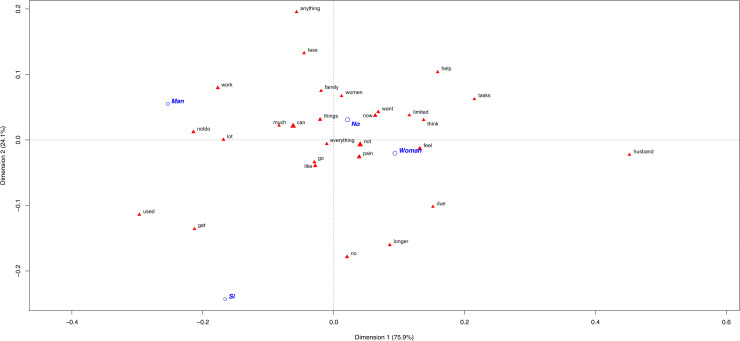
Most frequently used words in CNCP patients’ narrative according to OUD diagnosis and sex. The first plane of the correspondence analysis with the most contributive terms of the Chronic Non-Cancer Pain (CNCP) patients due to Opioid Use Disorder (OUD) according to sex (male, female). The different categories appear in blue: DSM-5 diagnosis (yes =  DSM-5 Si/ no =  DSM-5 No), sex (male =  man/ female =  woman).

More significant words were used by the no-OUD individuals, from which sex-related differences can be remarked. The most significant word for the no-OUD women was *“husband”*, followed by *“tasks”* (Fig 2). Some explained that: *“When I can’t do housework, I rest and then continue. My husband does nothing at home”, “I must make meals because my husband works late, and someone has to feed our children”* and *“I can’t do house tasks. We hired someone to clean the house. My husband supports me”*. In contrast, men’s most discriminatory word was *“work”* (Fig 2). They mentioned *“Work disability”* numerous times, and they explained that *“I can no longer perform the same at work”, “I have had to leave work”* and *“I can no longer work”*. The next significant word was *“anything”* and was used in the context of: *“I don’t feel like doing anything”, “If this progresses, I won’t be able to do anything”* and *“I’m useless, I can’t do anything”*.

#### Gender productive role.

**Fig 3:**
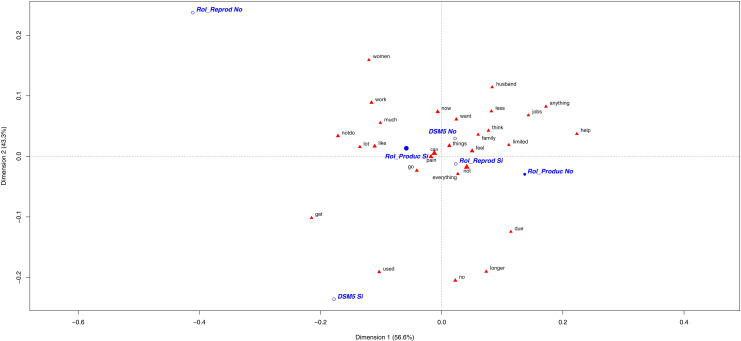
Most frequently used words in CNCP patients’ narrative according to OUD diagnosis and gender role. The first plane of the correspondence analysis with the most contributive terms of the Chronic Non-Cancer Pain (CNCP) patients due to Opioid Use Disorder (OUD) according to gender role (productive, reproductive). The different categories appear in blue: DSM-5 diagnosis (yes =  DSM-5 Si/ no =  DSM-5 No), productive role (yes =  Rol_Produc Si/ no =  Rol_Produc No), reproductive role (yes =  Rol_Reprod Si/ no =  Rol_Reprod No).

The most significant word of the patients with productive roles was *“women”* (Fig 3). It was used mainly by productive women to describe themselves; some sample testimonials included: *“Women have a heavier family, work and social burden. More workload/having to ensure pain”*, *“Women are much more resilient”* and *“Because my husband’s activity is not the same as mine in terms of housework and children, I think it has a stronger impact on women”.* Most men with this same role used *“women”* to support the stereotypical idea of what women are supposed to do and be, as follows: *“I think that women shoulder more responsibilities; house, family, work”* and *“I have certain home obligations that women tend to take on, which I have to perform despite pain”*.

The following most significant word was *“work”*, which was employed mainly by men in testimonials such as *“I had to stop working because of my limitations”* and *“I’ve had to stop working and ask for disability”*. These speeches suggest that men tend to play more productive roles than women and focus on work and economical situations. For the women with a productive role, they used this word to refer to housework: *“Now someone comes to do the hard work and I do lighter jobs”* and *“Sometimes I can’t do housework”*.

#### Gender reproductive role.

The patients with reproductive roles shared similarities with women’s statements, and the most discriminatory words were *“husband”, “anything”* and *“help”* (Fig 3). Some women with this role described their experience with these words as *“ I´m devastated because I can’t do activities for my family. My husband helps me a lot”, “I can’t do housework. We hired someone to clean the house. My husband supports me”* and *“I can’t do anything; I feel limited to do anything and powerless”*. In contrast, very few men with a reproductive role used the word *“husband”*, which indicates that this role is characteristic of women. Some sample testimonials were *“I feel useless because I can’t do anything and need help to do everything”, “I’d like to do things, but I feel helpless because I can’t”* and *“Now as I can’t do anything or go out, I use my mobile phone”.* These were very similar to those of the women with this role.

### Economic impact on the CNCP and OUD patients

#### Copayment impact.

Regardless of patients’ economic status, those with OUD still did not have a broad lexicon. Therefore, very few differences were observed between them and the control patients. The low and high copayment patients with OUD diagnosis used different words to explain how pain had influenced their lives, but shared one message: *“due to pain, they can no longer do much or what they used to do/be”*. Some testimonials to illustrate this were *“I don’t do it because of pain and lack of desire”, “I can no longer do the same activities. I’m more discouraged”* and *“Everything has changed me because I’m no longer who I used to be. I did certain things before, but not now”.*

**Fig 4:**
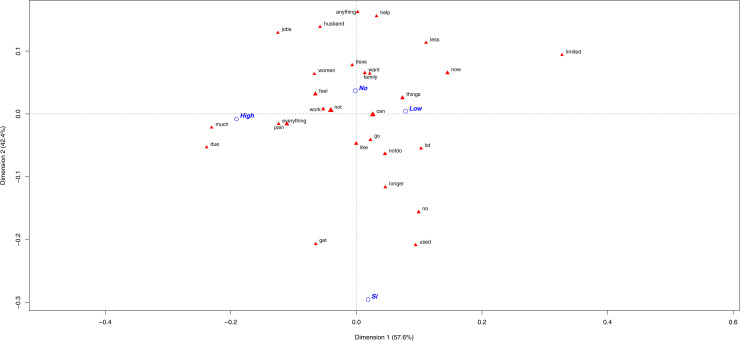
Most frequent words used by CNCP groups of patients according to their copayment percentage. The first plane of the correspondence analysis with the most contributive terms of the Chronic Non-Cancer Pain (CNCP) patients according to their copayment percentage classified as: a low copayment, those who have to pay <  40% of prescribed medications; or high copayment, those who pay ≥  40% of the total price of the prescribed drugs. The different categories appear in blue: CNCP diagnosis (yes =  Si/ no =  No), low copayment (Low), high copayment (High).

With the no-OUD patients, more differences driven by their copayment percentage were observed. The word *“limited”* was the most discriminating one for those with a low copayment percentage, followed by *“less”* and *“help”* (Fig 4). These patients indicated *“I´m limited to everything”, “Because I can’t do many things anymore and I look helpless”, “I feel I’m powerless”, “I’m useless”* and *“Having to depend on others makes me feel bad. I need help to do things at home”.* The patients with a high copayment percentage were characterized by the words *“tasks”* and *“work”*. They were more worried about: *“As I can’t do many tasks, I need help”, “I can’t keep on top of tasks like before”, “I’m not able to work to do anything. My position has changed a lot, at all levels”* and *“I really loved working, but I’ve given up”*.

## Discussion

The text mining analysis showed that the OUD chronic pain patients used a simpler lexicon with words that did not provide clear information about how they described their pain, and with no sex, gender or economic status significant differences. In contrast, the no-OUD CNCP subjects used richer vocabulary and terms to describe functional limitations caused by pain, especially for the low copayment percentage patients with the words “less” and “help”, and also with sex and gender clear differences. Speeches also suggested that the CNCP patients with a self-reported reproductive gender role used the same lexicon between men and women, which had a stronger impact of gender role than sex. Here men tended to play more productive roles than women and to focus on “work” and economical situations. In contrast, reproductive roles stressed the words “husband”, “anything” and “help”, but with no sex differences. Understanding and addressing social health determinants between genders is an essential first step towards achieving an appropriate pain management.

Results indicate the need for health professionals to act more intentionally by applying an intersectional lens when identifying priorities in OUD pain management [44,45]. Probably the scarce OUD sample avoids finding a relevant sex and gender impact in narratives. In this sense, other authors have observed how OUD men exhibit significantly lower distress tolerance levels than OUD women and men with no history of opioid dependence [46]. Thus, the OUD’s poor lexicon in our study should be more profoundly analysed to understand the social base of sex- differences observed in OUD studies in our region [47]. Moreover, according to our results, a stronger impact on mental health was observed for women in both groups, together with a significant difference in age and employment status. This highlights the need for further research into the role of biological sex in opioid use to prevent potential health inequities in OUD prevalence and management [41]. Its future implementation in clinical guides to prevent OUD in long-term opioid users is essential, especially for primary care providers who are at the forefront of the efforts for its prevention during routine screening of CNCP patients [48].

Literature findings suggest that women are more likely to be prescribed opioids for non-medical use, and they often present higher emotional and affective distress compared to men [49]. Our chronic pain women had experienced a sense of a lack of understanding by others when explaining their inability to continue with their work and home responsibilities [50,51]. This is relevant because, although household tasks may have changed in past decades, significant gender pain inequalities still exist [52]. Our female sample were still responsible for domestic work, working double and triple shifts, and received very little support in care tasks, which have been associated with poorer health [53]. About being a homemaker, our study evidenced that OUD patients were associated with higher unemployment rates and women were the only homemakers. In fact 34% of the included subjects mentioned reproductive roles, which shared similarities with women’s statements, and the most discriminatory words were “husband”, “anything” and “help”. In contrast, previous data evidenced that unemployment status was considered a relevant indicator of pain-related disability in men [54]. In resume, results suggests that these women might be exposed to a heavier family burden, which would stress the female lifetime, and their heavier working domestic and occupational burdens, which may imply a higher risk of developing painful disease [51] being a source of pain stereotypes and potential opioid use biases [55]. Advancing pain research requires clear and transparent reporting on sex/gender inequities, including younger CNCP patients (under 45-50 years old) in our samples to compare our data to, and working towards greater gender equality at home to improve women’s health and well-being [53,56]. All this information could help clinicians to better respond to the psychological health needs of women in the CNCP population in OUD deprescription procedures [41,57].

Of course, the social context and determinants are also relevant features of social learning approaches, which used to be applied, separately, to gender role development and pain being paid far less analysis than clinical characteristics [58,59]. In this sense, the text mining analysis has been a great help to understand this intersectional perspective with drug-copayment in OUD narratives. Previous data revealed that people with low socioeconomic status are at higher risk of developing long-term opioid use [60] In our region, different socio-economic statuses between the no-OUD subjects were intersected by sex [61] The fact that 91% of our population has monthly incomes below 1,000 euros, which corresponds to the minimum wage in Spain, limited our analysis in OUD subjects. It is important to note that the OUD group was smaller than the controls due to the low prevalence of dependent cases in PU in the real world. Anyhow, more knowledge of the socio-economic risk factors for long-term pain is necessary to facilitate effective communication during pain treatment [24,54].

In this sense, we propose three research areas that should be prioritized for an equal pain care: (1) Improve the understanding of gender differences in CNCP management even more due to the stronger impact on women’s mental health; (2) Consider socioeconomic status in opioid long use for chronic pain, and work towards a greater gender equality in reproductive roles as non-paid work; (3) Conduct sex and gender-stratified clinical trials with a representative sample of women and men.

## Limitations

There are some limitations in this study that need to be acknowledged. Firstly, the sample size was limited by a “convenience sample” with similar demographics (white, middle-aged) who came from a single hospital and were predominantly women. Consequently, the results of this study may not represent a broader population’s experience. In addition, the OUD patients provided very few narratives to explain their pain experience, which limits the extent of the qualitative analysis between patient groups. Secondly, the employed gender questionnaire is a new scale that has to be validated in other heterogeneous populations to assess its utility. Thirdly, given our sample’s relatively older mean age, the number of patients who actively worked was very small, and sex differences were difficult to compare together with the discussion of gender roles. As there are not enough younger (under 45 or under 50) people to compare to, this would be worth studying in the future. Furthermore, the financial situation can be a variable that might change along the patients’ lifespan, which was not considered in the present study. Finally, there are other important factors that were not controlled for in this study, such as pain duration, family/social support, or non-binary subjects. As patients could be classified with both gender roles, very few testimonials were available for the text mining analysis for those who played only one of the roles. Surprisingly, OUD’s quality of life and socialisation were better than for no-OUD despite their simpler lexicon. All this should be considered in future analyses as well as performing more complex text mining analysis [62–64]. Given the differences shown, we will continue to work with Natural Language Processing, and analysis of the emotional impact of content. All of these would help to understand this OUD. complex population that used to come with lived childhood experiences, a variety of very different pain diagnoses, as well as a vast range of mental health diagnoses and psychosocial histories.

Opioids, known for their potent pain-relieving characteristics, are essential in pain management, but they come with substantial drawbacks such as dependence. Our findings show a strong influence of OUD on narratives, together with a significant impact of sex, gender roles and economic status on CNCP. Moreover, pain-related gender stereotypes may still limit early and equal pain care access for women with a stronger impact on their mental health. These data highlight the need for an interdisciplinary patient management with CNCP even more when an OUD has been diagnosed.

## Supporting information

S1 Table Significant differences by sex for the CNCP patients due to OUD diagnosis (DSM-5V yes/no). Values are mean (SD), median [IQR] or %.
(DOCX)

S2 Table Differences in IDF-R between CNCP patients due to OUD diagnosis (DSM-5 yes/no). Values are mean (SD). No significant sex-differences were found. Here only a tendency was obtained.
(DOCX)

S3 Table Gender roles of the CNCP patients and comparison by sex. Values represent the percentage of patients with different gender roles grouped by DSM-5 and Sex (% is calculated by rows).
(DOCX)

S4 Table Important terms definitions. (DOCX)

S1 Questionnaire Gender questionnaire obtained from the CNCP patients. (DOCX)

S1 Fig Conceptual framework of gender and sex. Adapted from Gupta GR et al. Gender Equality, Norms, and Health Steering Committee. Gender equality and gender norms: framing the opportunities for health. Lancet. 2019;393(10190):2550-2562.
(PDF)
