# Supplementary material for: The silence of opioids-dependent chronic pain patients: A text mining analysis from sex and gender perspective
Source: PLoS One. 2025 Mar 18;20(3):e0319574. doi: 10.1371/journal.pone.0319574 (PMC11918440; doi:10.1371/journal.pone.0319574)
Supplement: S1 Table — Values are mean (SD), median [IQR] or %. (DOCX) [file pone.0319574.s001.docx]

**S1 Table. Significant differences by sex for the Chronic Non-Cancer Pain (CNCP) patients due to Opioid Use Disorder (OUD) diagnosis (DSM-5 yes/no).**

|  | OUD | | no-OUD | |
| --- | --- | --- | --- | --- |
| Outcomes (mean (SD)) | **Men**  (n= 10) | **Women**  (n= 22) | **Men**  (n= 59) | **Women**  (n= 147) |
| Age (years) | 57 (8) | 67 (13) | 58 (13) | 64 (14) |
| Employment Status (%) |  |  |  |  |
| Working | 0 | 9 | 20 | 17 |
| Retired | 40 | 32 | 41 | 33 |
| Work disability | 30 | 14 | 29 | 20 |
| Unemployed | 20 | 18 | 5 | 10 |
| Homemaker | 0 | 9 | 0 | 21 |
| MCS (SF12, 0-100) | 35 (12) | 39 [27) | 48 [23) | 40 [17) |
| Anxiety (HADS, 0-21) | 6 (1) | 12 (5) | 7 [5) | 8 (6) |
| Negative | 40 | 14 | 41 | 36 |
| Doubt | 0 | 9 | 12 | 16 |
| Case | 0 | 32 | 15 | 21 |
| Antidepressants | 6 | 6 | 61 | 39 |

*Values are mean (SD), median [IQR] or %. MCS: Mental component score; SF12: Short Format Health Survey 12; HADS: Hospital Anxiety and Depression Scale. In bold: sex related differences. In bold and grey: differences between the OUD and no-OUD participants.*
