# Supplementary material for: The silence of opioids-dependent chronic pain patients: A text mining analysis from sex and gender perspective
Source: PLoS One. 2025 Mar 18;20(3):e0319574. doi: 10.1371/journal.pone.0319574 (PMC11918440; doi:10.1371/journal.pone.0319574)
Supplement: S2 Table — Values are mean (SD). No significant sex-differences were found. Here only a tendency was obtained. (DOCX) [file pone.0319574.s002.docx]

**S2 Table. Differences in the Impairment and Functioning Inventory (IDF-R) scale between Chronic Non-Cancer Pain (CNCP) patients due to Opioid Use Disorder (OUD) diagnosis (DSM-V yes/no).**

|  | TOTAL  (n= 238) | OUD  (n= 32) | no-OUD  (n= 206) |
| --- | --- | --- | --- |
| IDF-R |  |  |  |
| Level functioning | 57 (38) | 58 (37) | 57 (39) |
| Household activities | 21 (21) | 18 (24) | 21 (21) |
| Independent functioning | 17 (8) | 19 (10) | 17 (9) |
| Social activities | 10 (7) | 12 (4) | 9 (6) |
| Leisure activities | 10 (10) | 11 (6) | 10 (10) |
| Impairment level | 5 (9) | 6 (10) | 5 (8) |

*Values are mean (SD). No significant sex-differences were found. Here only a tendency was obtained. IDF-R: Impairment and Functioning Inventory scale. The tendency values are shown in bold.*
