# Supplementary material for: The silence of opioids-dependent chronic pain patients: A text mining analysis from sex and gender perspective
Source: PLoS One. 2025 Mar 18;20(3):e0319574. doi: 10.1371/journal.pone.0319574 (PMC11918440; doi:10.1371/journal.pone.0319574)
Supplement: S3 Table — Values represent the percentage of patients with different gender roles grouped by DSM-5 and Sex (% is calculated by rows). (DOCX) [file pone.0319574.s003.docx]

**S4 Table. Gender roles of the Chronic Non-Cancer Pain (CNCP) patients and comparison by sex.**

|  |  | **Gender Roles** | | |
| --- | --- | --- | --- | --- |
| **Outcomes** (%) | | **Productive** | **Reproductive** | **Both Roles** |
| **DSM-5 diagnosis** |  |  |  |  |
|  | OUD *(n=32)* | 6 | 28 | 47 |
|  | no-OUD *(n=206)* | 4 | 34 | 51 |
| **Sex** |  |  |  |  |
|  | Male *(n=69)* | 10 | 22 | 59 |
|  | Female *(n=169)* | 2 | 38 | 47 |
|  | Total *(n=238)* | 5 | 34 | 51 |

*Values represent the percentage of patients with different gender roles grouped by DSM-5 and Sex (% is calculated by rows). In bold and grey: significant differences when comparing gender roles in groups of patients based on the DSM-5 criteria (OUD vs. no-OUD) and sex (Male vs. Female).*
