# Supplementary material for: The silence of opioids-dependent chronic pain patients: A text mining analysis from sex and gender perspective
Source: PLoS One. 2025 Mar 18;20(3):e0319574. doi: 10.1371/journal.pone.0319574 (PMC11918440; doi:10.1371/journal.pone.0319574)
Supplement: S4 Table — (DOCX) [file pone.0319574.s004.docx]

**S5 Table. Important terms definitions.**

| **Outcome** | **Definitions** |
| --- | --- |
| Sex | Biological differences between women and men, specifically reproductive organs and their functions. These sets of biological characteristics are not mutually exclusive, as there are individuals who possess both, but these characteristics tend to differentiate humans as women or men [1-4]. |
| Gender | Social context in which people live and which contributes to a subjective sexual identity, masculine or feminine, implying constructed roles, behaviors, expressions and identities of girls, women, boys, men, and gender diverse people i.e. **gender identity** is not confined to a binary (girl/woman, boy/man) nor is it static; it exists along a continuum and can change over time. When individuals or groups do not “fit” established gender norms they often face stigma, discriminatory practices or social exclusion, all of which adversely affect health as **gender stereotypes** [2-7]. |
| Gender norms | A complementary but separate term, refers to the explicit and implicit rules that govern the acceptable expression and behaviors for males and females within the family, community, workplaces, and wider society.  Gender norms are impressed upon individuals, thus shaping and modeling people’s values and options in the world i.e. **gender roles** (productive: paid-workers, usually applied to males; and reproductive: individuals overseeing domestic tasks, usually applied to females (childcare, parenting assistance, cooking, cleaning). It influences how people perceive themselves and each other, how they act and interact, and the distribution of power and resources in society. Other important criteria for sociocultural analysis include class, race, poverty level, ethnic group and age [8]. |

1. Colineaux H, Soulier A, Lepage B, Kelly-Irving M. Considering sex and gender in Epidemiology: a challenge beyond terminology. From conceptual analysis to methodological strategies. Biol Sex Differ. 2022 May 12;13(1):23. doi: 10.1186/s13293-022-00430-6. PMID: 35550193; PMCID: PMC9103114.

2. King BM. Point: a call for proper usage of "gender" and "sex" in biomedical publications. Am J Physiol Regul Integr Comp Physiol. 2010 Jun;298(6):R1700-1. doi: 10.1152/ajpregu.00694.2009. Epub 2010 Mar 31. PMID: 20357018.

3. Hammarström A, Johansson K, Annandale E, Ahlgren C, Aléx L, Christianson M, Elwér S, Eriksson C, Fjellman-Wiklund A, Gilenstam K, Gustafsson PE, Harryson L, Lehti A, Stenberg G, Verdonk P. Central gender theoretical concepts in health research: the state of the art. J Epidemiol Community Health. 2014 Feb;68(2):185-90. doi: 10.1136/jech-2013-202572. Epub 2013 Nov 21. PMID: 24265394.

4. Sex and gender - Gender Matters - <https://www.coe.int/en/web/gender-matters/sex-and-gender#17> Gender Matters. Data published December 02, 2022. Data accessed December 3, 2024.

5. What is gender? What is sex? Government of Canada. <https://cihr-irsc.gc.ca/e/48642.html> CIHR. Data published May 08, 2023. Data accessed December 3, 2024.

6. Informed by Gender Equality Glossary and Thesaurus, by European Institute for Gender Equality, n.d.; Integrating Gender Perspective into Statistics, by United Nations Statistical Division, n.d.; & by Gender Equality Glossary, COUNCIL OF EUROPE. (2016, revised in 2022)

7. Gender Equality. Glossary of Terms and Concepts. UNICEF Regional Office. (2017)

8. Gender Equality, Norms & Health – Gary Darmstadt. <https://genderandhealthcommission.org/blog/gender-equality-norms-health-gary-darmstadt/> Gender Equality, Norms & Health – Gary Darmstadt | The Lancet Commission on Gender and Global Health. Data accessed December 3, 2024.
