# Supplementary material for: The silence of opioids-dependent chronic pain patients: A text mining analysis from sex and gender perspective
Source: PLoS One. 2025 Mar 18;20(3):e0319574. doi: 10.1371/journal.pone.0319574 (PMC11918440; doi:10.1371/journal.pone.0319574)
Supplement: S1 Questionnaire — (DOCX) [file pone.0319574.s005.docx]

**S3 Questionnaire. Gender questionnaire obtained from the Chronic Non-Cancer Pain (CNCP) patients.**

1. Has your pain changed the way you are? Yes/No. How?
2. Has pain affected your self-esteem as a woman/man? Yes/No. How?
3. Has pain changed your image of yourself as a man/woman? Yes/No. How?
4. Has pain changed your masculinity or femininity? Yes/No. How?
5. Has pain generated a conflict between what you want/can (do) and what you think your family environment expects of you as a woman/man? Yes/No. How?
6. Has pain generated a conflict between what you want/can (do) and what the social environment asks of you as a woman/man? Yes/No. How?
7. Has pain affected your work tasks and/or responsibilities in your work environment?

Yes/No. How?

1. Did you do household tasks before being diagnosed with the disease? Yes/No.
2. Has pain affected your tasks and/or domestic responsibilities? Yes/No. How?
3. Has pain affected your life project or your future plans? Yes/No. How?
4. Has pain affected your relationships? Yes/No. How?
5. Has pain affected your sexual relations? Yes/No. How?
6. Has pain affected your family relationships? Yes/No. How?
7. Do you think that your social, work or family position has worsened due to pain?

Yes/No. How?

1. Do you think that pain experience would have been different instead of a man being a woman (or vice versa)? Yes/No. How?
