# Supplementary material for: The silence of opioids-dependent chronic pain patients: A text mining analysis from sex and gender perspective
Source: PLoS One. 2025 Mar 18;20(3):e0319574. doi: 10.1371/journal.pone.0319574 (PMC11918440; doi:10.1371/journal.pone.0319574)
Supplement: S1 Fig — Adapted from Gupta GR et al. Gender Equality, Norms, and Health Steering Committee. Gender equality and gender norms: framing the opportunities for health. Lancet. 2019;393(10190):2550-2562. (PDF) [file pone.0319574.s006.pdf]

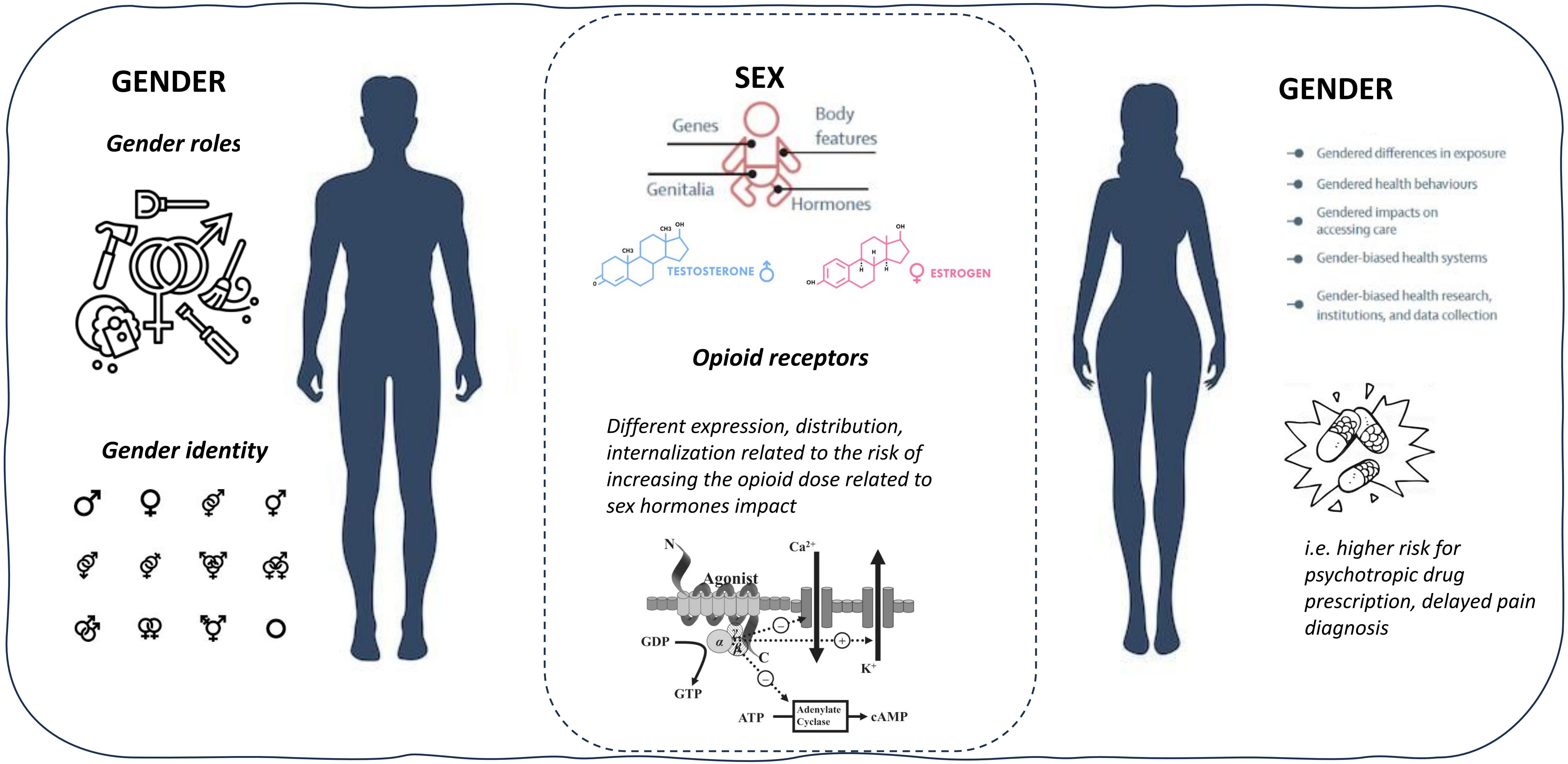
GENDER
Gender roles
Gender identity
SEX
Genes
Body features
Genitalia
Hormones
TESTOSTERONE
ESTROGEN
Opioid receptors
Different expression, distribution, internalization related to the risk of increasing the opioid dose related to sex hormones impact
N
Agonist
Ca^{2+}
GDP
GTP
C
K^{+}
ATP
Adenylate Cyclase
cAMP
GENDER
Gendered differences in exposure
Gendered health behaviours
Gendered impacts on accessing care
Gender-biased health systems
Gender-biased health research, institutions, and data collection
i.e. higher risk for psychotropic drug prescription, delayed pain diagnosis
